# Inhibition of Jak-STAT3 pathway enhances bufalin-induced apoptosis in colon cancer SW620 cells

**DOI:** 10.1186/1477-7819-10-228

**Published:** 2012-10-30

**Authors:** Zhitu Zhu, Enze Li, Yangyang Liu, Yu Gao, Hongzhi Sun, Guangyou Ma, Zhenghua Wang, Xiaomei Liu, Qingjun Wang, Xiujuan Qu, Yunpeng Liu, Yunlong Yu

**Affiliations:** 1 Department of Oncology, The First Affiliated Hospital of Liaoning Medical University, Jinzhou, Liaoning, 121001, China; 2 Department of Oncology, The First Affiliated Hospital of Chinese Medical University, No 155, Nanjingbei Street, Heping District, Shenyang, Liaoning, 11001, China; 3 Department of Oncology, China National Offshore Oil Corporation Hospital, No 10, Shiyou Rd, Tanggu District, Tianjing, 300452, China; 4 Department of Oncology, The First Affiliated Hospital of Liaoning Medical University, No 2, Wuduan Rengmin Street, Jinzhou, 121001, China

**Keywords:** Colon cancer, Jak-stat3, Bufalin, Livin, Caspase, M-phase arrest, Apoptosis

## Abstract

**Background:**

The purpose of the research is to investigate the roles of Jak-STAT3 signaling pathway in bufalin-induced apoptosis in colon cancer SW620 cells.

**Methods:**

The inhibitory effects of bufalin on cell proliferation were determined by MTT (Methyl thiazolyltetrazolium) assay. The morphological changes of cells were measured by Wright-Giemsa staining. The cell cycle arrest and apoptosis were tested by flow cytometry analysis. Western Blot was used to determine the protein expression of the apoptosis inhibitors livin and caspase-3, the apoptosis-related proteins Bax and Bcl-2, as well as the key protein kinases in the Jak-stat3 signaling pathway, stat3 and p-stat3.

**Results:**

(1) Bufalin inhibited the proliferation of SW620 cells. IC50 at 24 h, 48 h and 72 h were 76.72 ± 6.21 nmol/L, 34.05 ± 4.21 nmol/L and 16.7 ± 6.37 nmol/L. (2) Bufalin induced SW620 cell cycle arrest and apoptosis, indicated by the appearance of apoptotic bodies; (3) The results from flow cytometry demonstrated that there was cell cycle G2/M phase arrest in 20 nmol/L bufalin treatment group (36.29 ± 2.11% vs 18.39 ± 1.74%, P<0.01); there was a sub-diploid apoptosis peak in 80 nmol/L bufalin treatment group (19.69 ± 1.63% vs 0.99 ± 0.23%, P <0.01). The apoptosis rate was 34.63 ± 2.57% (vs 19.69 ± 1.63%, P = 0.002) in JAK kinase inhibitor AG490 plus bufalin treatment group. (4) During the process of bufalin-induced apoptosis in SW620 cells, transient activation of p-stat3 inhibited the activation of stat3, up-regulated Bax expression, down-regulated livin and Bcl-2 expression (P<0.01), and activated caspase-3. Inhibition of Jak-stat3 signaling pathway by pre-treatment with AG490 significantly enhanced the bufalin-induced apoptosis (P<0.01), further up-regulated Bax protein expression, down-regulated livin and Bcl-2 protein expression and enhanced caspase-3 activation.

**Conclusions:**

Bufalin not only inhibited the growth of colon cancer SW620 cells, but also induced apoptosis of SW620 cells. Activation of caspase-3, up-regulation of Bax, down-regulation of livin and Bcl-2, as well as inhibition of Jak-stat3 signaling pathway might be the important mechanisms for the bufalin-induced apoptosis.

## Background

Colon cancer is a common digestive system cancer. It is estimated that there were 106,100 new cases and 49,920 deaths from colon cancer in the US in 2009 [1]. Due to the change in people’s diet, the incidence of colon cancer is rising and becoming one of the most common malignancies and the leading cause of death from cancer. Surgical resection remains the most common treatment for colorectal cancer; however, by the time people are diagnosed the cancer may be more advanced, so chemotherapy-based comprehensive treatment is a crucial means for colon cancer treatment. In recent years, with the development and application of a new generation of chemotherapy and molecular-targeted drugs, the effects of colorectal cancer treatment have been improved, but are still unsatisfactory. In addition, the toxic side effects of chemotherapy drugs and the failure of chemotherapy due to drug resistance are some of the drawbacks of clinical treatment [2]. Therefore, researchers are engaged in discovering effective and low toxicity drugs for colon cancer treatment to improve the cure rate and reduce mortality of colon cancer.

Bufalin is isolated from the secretion of the skin and parotid venom glands of the Chinese toad (*Bufobufogargarizans cantor*) and black-spectacled toad (*Bufomelanostictus*) and is the major digoxin-like immunoreactive component of the traditional Chinese medicine, *Chan Su*. Bufalin is a cardiotonic steroid with the molecular formula C_24_H_34_O_4_ and molecular weight of 386.5 [3]. Studies show that bufalin can effectively inhibit the proliferation of various tumor cells [4]. It has been shown that bufalin-induced leukemia cell apoptosis down-regulates Bcl-2, survivin and WT1, activates PKCbII and caspase-3, and triggers release of Smac/DIABLO from mitochondria [5-7]. Watabe *et al*. [8] reported that persistent activation of extracellular-regulated protein kinases (ERK) is an important mechanism of bufalin-induced apoptosis in human leukemia U937 cells. However, the mechanisms of bufalin-induced apoptosis remain elusive. Moreover, there are few studies on the roles of bufalin in the treatment of colon cancer.

Digestive system cancer is a common clinical cancer and it is an accumulative process that involves multiple factors and multiple mutations. The etiology of the cancer remains elusive. The studies on the over-expression or abnormal activation of the proteins that relate to the occurrence, development and migration of the cancers have recently become hot topics. Much attention has been focused on the relevance between the signal transducers and activators of transcription 3 (STAT3) and digestive cancer. STAT3 is a member of the signal transducers and activators of transcription (STATs) family [9], which is a protein family that presents in the cytoplasm and can be activated by phosphorylation and transferred into the nuclei to bind DNA, and performs a dual function with respect to signal transduction and transcriptional regulation. In the STATs family, STAT3 is much more prone to have structural phosphorylation and trigger tumorigenesis. Studies show that STAT3 are constitutively activated and abnormally expressed in tumor tissues and cell lines, and promote tumor proliferation, differentiation, invasion, metastasis, angiogenesis and immune escape by modulating the downstream genes livin and caspase-3 [10]. Scheper *et al*. [11] have shown that inhibition of STAT3 activation may reduce survivin expression in gastric cancer and oral squamous cell carcinoma cell lines and promote apoptosis of cancer cells. In addition, stable transfection and expression of survivin in cells could reverse the stat3-inhibitor-induced pro-apoptotic effects [11,12]. Livin and survivin belong to the inhibitor of apoptosis proteins (IAP) family; however, it is still unclear whether STAT3 can affect livin expression. Studies show that many cytotoxic drugs induce tumor cell apoptosis by inhibiting STAT3, indicating STAT3 might be a new target for cancer therapy [13]. It still remains unclear whether STAT3 plays a role in bufalin-induced apoptosis in cancer cells, especially colon cancer cells.

In this study we demonstrate that the Janus family tyrosine kinase (Jak)-STAT3 pathway modulates bufalin-induced apoptosis in SW620 colon cancer cells and affects livin, caspase-3, BCL2-associatedX protein (BAX) expression. This study further elaborates the mechanisms of bufalin-induced apoptosis in colon cancer cells and explored potential biological targets and signaling pathways.

## Methods

### Cell culture

Human colon cancer SW620 cell line was purchased from the Institute of Cell Biology, Chinese Academy of Sciences (Shanghai, China). SW620 cells were grown in RPMll640 culture medium containing 10% heat-inactivated fetal bovine serum, 100 U/mL penicillin and 100 mg/mL streptomycin. Cells were incubated in the humidified incubator at 37°C with 5% CO_2_, and passaged every 2 to 3 days by digestion with 0.25% trypsin. All experiments were performed using the log phase cells. This study was approved by hospital ethics committee.

### Reagents and antibodies

Mouse anti-human ERK and P-ERK antibody, mouse anti-human phosphor-STAT3 (p-STAT3), STAT3, poly (ADP-ribose) polymerase (PARP), BAX, BCL-2, livin and caspase-3 monoclonal antibodies were purchased from Santa Cruz Biotechnology (Santa Cruz, CA, USA). Propidium iodide (PI), RNase, methyl thiazolyltetrazolium (MTT), bufalinand AG490 were purchased from Sigma (St Louis, MI, USA). PD98059 was purchased from Promega (Madison, WI, USA). The bufalin was dissolved in 100% ethanol at a concentration of 0.01mol/L and stored at −20°C. The stock solution was diluted with culture media to the required concentration during experiments. The final ethanol concentration was less than 0.01%. The preliminary results showed that the concentration of ethanol did not affect cell proliferation.

### Methyl thiazolyltetrazolium assay

The log-phase cells were dissociated into a single cell suspension by enzyme digestion. The cell density was adjusted to 3 × 10^4^/mL and seeded in 96-well plates, 180 μL/well; 5, 10, 20, 40, 80 and 160 nmol/L of bufalin were added in the treatment groups and RPMI 1640 culture media were added in the control groups, and cultured for 24, 48 and 72 h. MTT 20 μL solution was added to each well 4 h before the termination of culture, and 200 μL dimethyl sulfoxide (DMSO) solution was added at the time of termination. After oscillation, the absorbance (A) values were measured at 490nm wavelength with a microplate reader. The proliferation inhibition rate (%) was calculated as follows:

$$\begin{array}{l} \text{Proliferation inhibition rate}\left(\%\right)\\ \;=1-\left(\text{Mean A value of treatment group}\right)\\ \;\left(-\text{Mean A value of blank control group}\right)/\\ \;\left(\text{Mean A value of the negative control group}\right)\\ \;\left(-\text{Mean A value of blank control group}\right)\times 100\%\text{.} \end{array}$$

The values of half-maximal inhibitory concentration (IC50) of bufalin were calculated. All experiments were repeated three times, mean values were calculated and the cell proliferation curves were drawn.

### Morphological staining and mitotic index determination

Cells were seeded in 6-well plates. The cell density was adjusted to 2.5 × 10^4^/well and incubated overnight. After 24 h reaction with 20 or 80 nmol/L bufalin, cells in negative control groups and treatment groups were collected, and centrifuged at 2000r/minute for 3 minutes. Cell smears were prepared and stained with Wright-Giemsa solution (Sigma) for 15 minutes, washed with running water and dried. The cell morphological changes were observed and photographed under a light microscope.

### Cell cycle phase and apoptosis analysis

Single staining: 1 × 10^6^ cells from control groups and treatment groups were collected and fixed with 70% cold ethanol at 4°C for 24 h, washed twice with cold PBS, and then RNase A (20 μg/mL) was added and incubated at 37°C for 30minutes. A final concentration of 10 g/L of PIwas added and reacted in the dark for 30 minutes. DNA content was measured by flow cytometry detection and the percentage of apoptosis was assayed and determined. Fluorescence2- Area (FL2-A) represented DNA content and the vertical axis represented cell number. CELL Quest software (Becton Dickinson, San Jose, CA, USA) was used for cell cycle analysis.

### Western blot

From control groups and treatment groups, 1 × 10^7^ cells were collected, homogenized with radioimmunoprecipitation assay (RIPA)lysis buffer (0.1% SDS, l% Triton-100, 150 mmol/L NaCl, 1 mmol/L EDTA (PH8.0), 10 mmol/L Tris -HCL (PH7.5)), incubated on ice for 30 minutes and centrifuged at 12000 r/minute for 30 minutes. The supernatants were harvested and the protein concentration per sample was determined using the Lowry method. The protein samples were mixed with 3 × sample buffer and boiled for 5 minutes. Protein (50 μg for each sample) was separated by 15% SDS-polyacrylamide gel electrophoresis for 3 h, and then transferred to nitrocellulose membranes. The membranes were blocked in 5% nonfat dried milk for 1 h and cut according to the pre-stained molecular weight markers. The membranes were incubated in the primary antibodies: p-ERK (1:1,000), ERK (1:500), p-STAT3 (1:1,000), STAT3 (1:1,000), PARP (1:1,000), BAX (1: 250), BCL-2 (1: 250), livin (1: 300), caspase-3 (1:1,000) and β-actin (1:1,000), overnight at 4°C. The membranes were washed by Tris-Tween Buffered Saline (TTBS) four times, and then incubated in horseradish peroxidase-conjugated secondary antibody (1:800) for 30 minutes at room temperature, visualized by ECL detection system, photographed and analyzed by the GIS gel image analysis system.

### Statistical analysis

All data were the results of three independent experiments and expressed as mean ± SD $\left(\overset{―}{x}\pm \text{s}\right)$. SPSS16.0 statistical software was used for one-way analysis of variance (ANOVA) and *t*-tests. Significance was placed at *P*<0.05.

## Results

### Bufalin inhibited the proliferation of colon cancer SW620 cells

**Figure 1:**
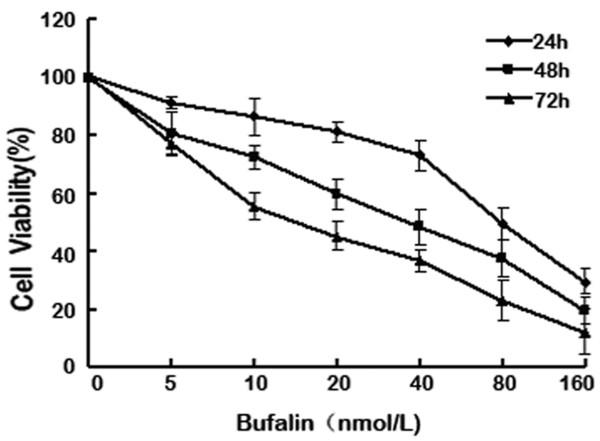
**Bufalin inhibited SW620 cell proliferation.** SW620 cells were exposed to bufalin (5 to160 nmol/L) for 24h, 48h and 72h, and cell viability was then determined by methyl thiazolyltetrazoliumassay (MTT). Data re the mean ± SD of at least three independent experiments performed in triplicate.

SW620 cells were exposed to 5 to 160 nmol/L bufalin for 24 h, 48 h and 72 h. MTT results showed that bufalin inhibited the proliferation of SW620 in a time- and dose-dependent manner (Figure 1). Results for IC50 at 24 h, 48 h and 72 h were 76.72 ± 6.21 nmol/L, 34.05 ± 4.21 nmol/L and 16.7 ± 6.37 nmol/L (Figure 1).

### Bufalin induced M-phase arrest and apoptosis in colon cancer SW620 cells

**Figure 2:**
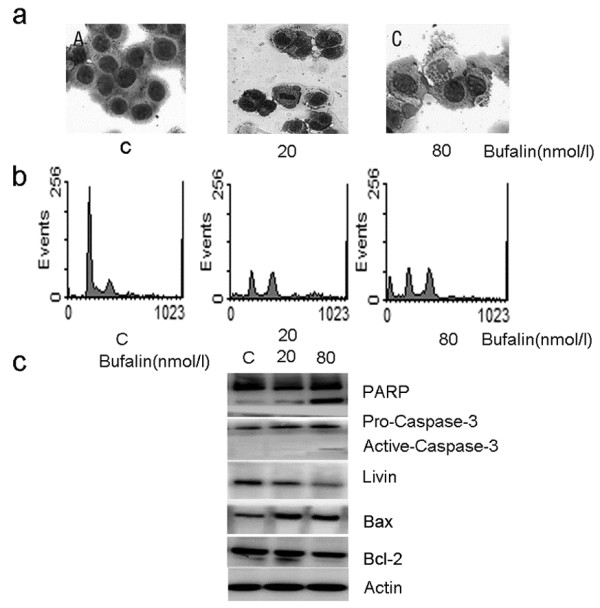
**Bufalin induced M-phase cell cycle arrest and apoptosis in SW620 cells.** Cells were exposed to 20 and 80 nmol/L bufalin for 24 h and then the cell morphological changes were determined by light microscopy (magnification×200) after Wright-Giemsa staining. (**a**) Cell cycle was analyzed by flow cytometry after staining with propidium iodide. (**b**) The expression of PARP, BAX, BCL-2, caspase-3 and livin proteins were analyzed by western blot. Actin was used as the internal control (**c**). Data shown are representative of one of three independent experiments.

SW620 cells were exposed to 20 nmol/L bufalin for 24 h. Light microscopy revealed an apparent G2/M-phase growth arrest: cells had two nuclei and stagnated in metaphase (Figure 2a). Flow cytometry analysis showed that the percentage of cells at G2/M phase was increased from 18.39 ± 1.74% (control group) to 36.29 ± 2.11% (treatment group) (*P*<0.001) (Figure 2b), indicating bufalin-induced M arrest in SW620 cells. Western blot showed no significant PARP cleavage, no caspase-3 activation, but a slight reduction in livin protein expression (Figure 2c). When SW620 cells were exposed to 80 nmol/L bufalin for 24 h, typical morphological features of apoptosis, such as apoptotic bodies, were observed (Figure 2a). There was an apparent hypodiploid apoptotic peak (sub-G1 peak) and the percentage of hypodiploid cells was 17.83 ± 2.38% vs. control group 1.24 ± 0.69% (*P*<0.001) (Figure 2b). Western blot showed significant PARP cleavage, appearance of active caspase-3 subunits and significant reduction in livin protein expression (Figure 2c). When SW620 cells were exposed to 20 nmol/L or 80 nmol/L bufalin for 24 h,western blot showed that in comparison with control groups, BAX expression increased 2.51- and 2.37-fold, respectively; BCL-2 expression decreased 87% and 74%, respectively; BAX/BCL-2 ratio increased 2.88- and 3.20-fold, respectively (Figure 2c). These results suggest that bufalin-induced apoptosis in SW620 cells is associated with the activation of caspase-3, down-regulation of livin and up-regulation of the BAX/BCL-2 ratio.

### Bufalin-induced apoptosis was enhanced by inhibition of Jak-STAT3 but not ERK

**Figure 3:**
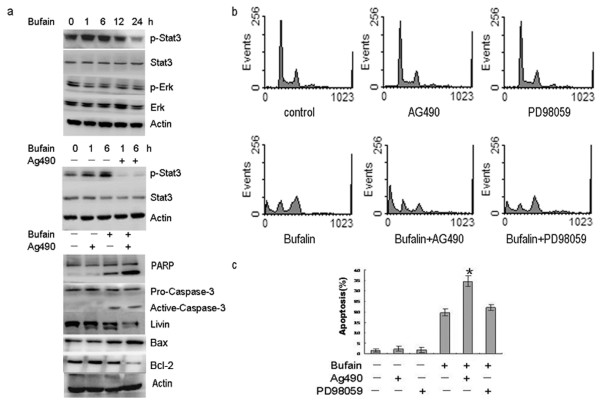
**Bufalin-induced apoptosis was enhanced by inhibition of Jak-STAT3 not ERK.** (**a**) Western blot detected the changes in the protein expression of p-STAT3, STAT3, P-ERK, ERK, PARP, BAX, BCL-2, caspase-3 and livin in SW620 cells that were exposed to 80 nmol/L bufalin alone or 80 nmol/L bufalin plus 20 μmol/L AG490. (**b**) Cells were exposed to 80 nmol/L bufalin for 24 h in the absence or presence of 20 μmol/L AG490 or 20 mmol/L PD98059. The cell cycle was analyzed by flow cytometry after staining with propidium iodide. The experiments were reproduced three times. One representative experiment is shown. (**c**) The percentage of apoptotic cells was analyzed by flow cytometry. *P< 0.05 vs. bufalin alone. Data are the mean ± SD of three independent experiments.

In order to verify the involvement of the Jak-STAT3 signaling pathway in bufalin-induced SW620 cells apoptosis, we measured the changes in the protein expression of STAT3 and phosphorylated stat3 (p-stat3) in SW620 cells that were exposed to 80 nmol/L bufalin for 1 to 24 h. Western blot results indicated that there was p-stat3 expression in control group SW620 cells. When SW620 cells were exposed to 80 nmol/L bufalin, p-STAT3 protein expression began to increase at 1 h, reached the peak at 6 h, began to decline at 12h and significantly decreased at 24 h. Expression of p-STAT3, in respect to control groups, were 161 ± 5.37% (1 h), 175 ±6.24% (6 h), 93.2 ± 4.49% (12 h) and 42.1 ± 5.73% (24 h). The total STAT3 protein levels did not change. There was no significant change in P-ERK and ERK protein expression (Figure 3a). When cells were pre-treated with 20 μmol/L AG490 (a selective inhibitor of the Jak pathway) for 1h and then exposed to 80 nmol/L bufalin for 1h and 6h, there was no p-STAT3 expression, suggesting that 20 μmol/L AG490 inhibited p-STAT3 activation (Figure 3a). When SW620 cells were exposed to 20 μmol/L AG490 alone for 24 h, Western blot results indicated that there was no PARP cleavage, no caspase-3 activation, and no significant reduction in livin, BAX and BCL-2 expression. When SW620 cells were pre-treated with 20 μmol/L AG490 for l h, and then exposed to 80 nmol/L bufalin for 24h, in comparison with bufalin alone, PARP cleavage was significantly enhanced, active caspase-3 subunits were increased, and livin protein expression was significantly decreased. BAX expression increased 3.22-foldvs. control group and 2.47-foldvs.bufalin alone; BCL-2 decreased 27% vs. control group and 62% vs.the bufalin alone group. In comparison with the control group, the BAX/BCL-2 ratio increased 3.98- and 11.92-fold in the bufalin alone and bufalin+AG490 groups, respectively (Figure 3a). When SW620 cells were exposed to 80 nmol/L bufalin for 24 h, flow cytometry analysis indicated that the apoptosis rate was 19.69 ± 1.63% , which was significantly higher than in the control group (*P*<0.01) (Figure 3b,c). Treatment with 20 μmol/L AG490 or 20 μmol/L PD98059 (a selective inhibitor of ERK) alone induced a small amount of SW620 cells apoptosis. However, when SW620 cells were pre-treated with 20 μmol/L AG490 or 20 μmol/L PD98059 for l h, and then exposed to 80 nmol/L bufalin for 24h, the apoptosis rate was increased from 19.69 ± 1.63% to 34.63 ± 2.57% (*P* = 0.002) and 22.17 ± 1.46% (*P* = 0.14), respectively. These results indicate that the Jak-STAT3, but not ERK, signaling pathway plays crucial roles in bufalin-induced apoptosis in SW620 cells.

## Discussion

Bufalin is isolated from the secretion of the skin and parotid venom glands of the Chinese toad (*Bufobufogargarizans cantor)* and the black-spectacled toad (*Bufomelanostictus*) and is the major component of *Chan Su*. In recent years, studies have showed that bufalin is an effective anti-cancer ingredient. Bufalin can inhibit the proliferation of HL-60 leukemia cells, ovarian cancer, gastric cancer and other tumor cells and induce cell cycle arrest and apoptosis [14-18]. The results from this study indicate that 20 nmol/L bufalin inhibited the proliferation of colon cancer SW620 cells and increased G2/M-phase cells, suggesting that 20 nmol/L bufalin induced cell cycle arrest; meanwhile, 80 nmol/L bufalin induced apoptosis. Studies show that bufalin induce leukemia cell apoptosis by activating cdc2 kinase, casein II, protein kinase A, and protein kinase C [19], AP-1 [20] and Rac1 [14]. Our results indicate that bufalin-induced colon cancer cell apoptosis is associated with the up-regulation of BAX, the down-regulation of livin and BCL-2, as well as the activation of caspase-3, indicating that bufalin induces apoptosis in different cells through different mechanisms.

Apoptosis is a genetically controlled programmed cell death and is an important mechanism for the body to maintain internal environment homeostasis. There are two pathways of apoptosis, one is the mitochondrial, and the other is the death receptor pathway. The function of the mitochondrial pathway in apoptosis has become a hot topic in recent years [21]. The mitochondrial pathway is regulated by multiple genes. The anti-apoptotic genes mainly include the BCL-2 gene family and the IAP family. Livin is an important member of the IAP family. Livin is mainly expressed in embryonic tissues and is absent in most normal tissues. However, livin expression can be detected in almost all malignant tumors [22]. Livin is closely associated with the occurrence, development, degree of malignancy, prognosis and drug resistance of tumors, and may become a new target for early diagnosis and treatment of cancers [23]. Livin can inhibit the pro-apoptotic factor SMAC-induced activation of the caspasefamily, which causes tumor cells to gain resistance toward apoptosis. Livin can also inhibit apoptosis by directly inhibiting the activation of caspase-3 and caspase-7 [24]. Caspase is a group of aspartate-specific cysteine proteases and is currently considered one of the most critical proteins in apoptosis induction. Caspase-3 is a downstream executioner of the cascade and can be activated by the activation of caspase-8, 9 and 12. The activation of caspase-3 can disrupt cell structures through destroying the nuclear lamina, leading to cell apoptosis [25]. The results from this study show that livin protein expression significantly decreased in the bufalin-induced apoptosis. The detection of the caspase-3 active 17KD subunit, an active (cleaved) caspase-3, in SW620 cells exposed to 80 nmol/L bufalin for 24 h, indicates that bufalin induces apoptosis in a caspase-3-dependent manner.

The mitochondrial apoptosis pathway is regulated by many factors. Research confirms that the ERK and Jak pathways are involved in the regulation of apoptosis and survival of tumor cells [26,27]. The Jak-STAT3signaling pathway is the major pathway that transfers membrane receptor signals to intracellular domains, and plays important roles in supporting cell survival and inhibiting apoptosis [28]. Recent studies demonstrate that the STAT3 signaling pathway is closely related to cell proliferation, differentiation and apoptosis. The activation pattern of STAT3 alters significantly in cancer cells, which is indicated by the abnormal expression of STAT3 and/or constitutive activation of the STAT3 signaling pathway. The change in the STAT3 activation pattern leads to abnormal cell proliferation or malignant transformation, so STAT3 is defined as a cancer gene. A variety of extracellular growth factors and inflammatory cytokines can induce the phosphorylation of stat3 to become activated p-STAT3. The level of activated p-STAT3 elevates in a variety of malignant tumors. In colon adenocarcinoma, p-STAT3 is an important factor associated with the extent of tumor invasion and poor prognosis [29,30]. Survivin is another important member of the IAP family. There are many similarities between survivin and livin. They are both intracellular proteins containing one or more baculovirus inhibitor of apoptosis protein repeat (BIR) domains and inhibit caspase activation [31]. Scheper and others [11,12] have shown that inhibition of STAT3 activation can reduce survivin protein expression in gastric cancer and oral squamous cell carcinoma cell lines, and promote apoptosis of cancer cells. The forced expression of survivin can reverse the pro-apoptosis effects induced by STAT3 inhibition [11,12]. In this study, we are the first to report that when colon cancer SW620 cells were pre-treated with 20 μmol/L Jak-STAT3 pathway inhibitor AG490 for 1 h, and then exposed to bufalin, the down-regulation of livin protein expression and activation of caspase-3 were enhanced, suggesting that livin and survivin might be STAT3 downstream targets. Other studies show that some cytotoxic drugs can induce apoptosis through the inhibition of the Jak-STAT3 signaling pathway [28]. To date, there are no reports available on the role of Jak-STAT3 signaling pathway in bufalin-induced apoptosis in colorectal cancer cells. In this study, the control group SW620 cells were expressing p-STAT3. When SW620 cells were exposed to 80 nmol/L bufalin for 1 to 24 h, p-STAT3 protein expression began to increase at 1h, reached the peak at 6 h, began to decline at 12 h, and significantly decreased at 24 h. The total STAT3 protein level did not change. When SW620 cells were pre-treated with 20 μmol/L AG490 for 1 h, and then exposed to 80 nmol/L of bufalin for 1 h and 6 h, p-STAT3 expression was not detected. In addition, inhibition of the Jak-STAT3 pathway enhanced the bufalin-induced apoptosis. Bufalin and the Jak-STAT3 signaling pathway inhibitor AG490 had a synergistic induction of apoptosis, suggesting that the Jak-STAT3 pathway plays an important role in bufalin-induced apoptosis in SW620 cells. There are contradictory reports on the function of the ERK pathway in bufalin-induced apoptosis. It has been reported that the abnormal activation of ERK pathway is required in bufalin-induced apoptosis in u937 cells [8]. Our results indicate that inhibition of the ERK pathway did not significantly affect the bufalin-induced apoptosis in SW620 cells. The discrepancy might be due to the different functions of the ERK pathway in different cells; it might also be related to cell-specific effects of bufalin. In conclusion, our study demonstrates that bufalin induces apoptosis in colon cancer cells through the inhibition of the Jak-STAT3 pathway. Future studies are geared toward evaluating the antitumor functions of bufalin in animal models.

## Conclusions

Bufalin not only inhibited the growth of colon cancer SW620 cells, but also induced apoptosis of SW620 cells. Activation of caspase-3, up-regulation of Bax, down-regulation of livin and Bcl-2, as well as inhibition of Jak-stat3 signaling pathway might be the important mechanisms for the bufalin-induced apoptosis.

## Abbreviations

BAX: BCL2-associated X protein; BIR: Baculovirus inhibitor of apoptosis protein repeat; DMSO: Dimethyl sulfoxide; ERK: Extracellular-regulated protein kinase; FL2-A: Fluorescence2- Area; IAP: Inhibitor of apoptosis proteins; IC: Inhibitory concentration; Jak: Janus family tyrosine kinase; MTT: Methyl thiazolyltetrazolium; PARP: Poly(ADP-ribose) polymerase; PBS: Phosphate-buffered saline; PI: Propidium iodide; RIPA: Radioimmunoprecipitation assay; STAT: Signal transducers and activators of transcription; TTBS: Tris-Tween Buffered Saline.

## Competing interests

No potential conflicts of interest are disclosed.

## Authors' contributions

ZZ and GM carried out the molecular genetic studies, participated in the sequence alignment and drafted the manuscript. HS, YG, and XL carried out the immunoassays. YY and YL participated in the sequence alignment. QW, ZW, and EL participated in the design of the study and performed the statistical analysis. XQ and YL conceived of the study, and participated in its design and coordination and helped to draft the manuscript. All authors read and approved the final manuscript.

## References

[B1] Jemal A Siegel R Ward E Hao Y Xu J Thun MJ Cancer statistics, 2009 CA Cancer J Clin 2009 59 225 249 10.3322/caac.20006 19474385

[B2] Peeters M Price TJ Cervantes A Sobrero AF Ducreux M Hotko Y André T Chan E Lordick F Punt CJ Strickland AH Wilson G Ciuleanu TE Roman L Van Cutsem E Tzekova V Collins S Oliner KS Rong A Gansert J Randomized phase III study of panitumumab with fluorouracil, leucovorin, and irinotecan (FOLFIRI) compared with FOLFIRI alone as second-line treatment in patients with metastatic colorectal cancer J Clin Oncol 2010 28 4706 4713 10.1200/JCO.2009.27.6055 20921462

[B3] Krenn L Kopp B Bufadienolides from animal and plant sources Phytochemistry 1998 48 1 29 10.1016/S0031-9422(97)00426-3 9621450

[B4] Cao-Hong Shibayama-Imazu T Masuda Y Shinki T Nakajo S Nakaya K Involvement of Tiam1 in apoptosis induced by bufalin in HeLa cells Anticancer Res 2007 27 245 249 17352239

[B5] Tian X Wang PP Liu YP Hou KZ Jin B Luo Y Qu XJ Effect of bufalin-inducing apoptosis on Bcl-2 and PKC in HL-60 cells Zhongguo Shi Yan Xue Ye Xue Za Zhi 2007 15 67 71 17490524

[B6] Liu Y Qu X Wang P Tian X Luo Y Liu S Lu X WT1 downregulation during K562 cell differentiation and apoptosis induced by bufalin Zhonghua Xue Ye Xue Za Zhi 2002 23 356 359 12411052

[B7] Tian X Luo Y Liu YP Hou KZ Jin B Zhang JD Wang S Downregulation of Bcl-2 and survivin expression and release of Smac/DIABLO involved in bufalin-induced HL-60 cell apoptosis Zhonghua Xue Ye Xue Za Zhi 2006 27 21 24 16732934

[B8] Watabe M Masuda Y Nakajo S Yoshida T Kuroiwa Y Nakaya K The cooperative interaction of two different signaling pathways in response to bufalin induces apoptosis in human leukemia U937 cells J Biol Chem 1996 271 14067 14072 10.1074/jbc.271.24.14067 8662906

[B9] Aggarwal BB Kunnumakkara AB Harikumar KB Gupta SR Tharakan ST Koca C Dey S Sung B Signal transducer and activator of transcription-3, inflammation, and cancer: how intimate is the relationship Ann NY Acad Sci 2009 1171 59 76 10.1111/j.1749-6632.2009.04911.x 19723038 PMC3141289

[B10] Huang S Regulation of metastases by signal transducer and activator of transcription 3 signaling pathway: clinical implications Clin Cancer Res 2007 13 1362 1366 10.1158/1078-0432.CCR-06-2313 17332277

[B11] Scheper MA Nikitakis NG Sauk JJ Survivin is a downstream target and effector of sulindac-sensitive oncogenic Stat3 signalling in head and neck cancer Int J Oral Maxillofac Surg 2007 36 632 639 10.1016/j.ijom.2007.04.003 17566705

[B12] Kanda N Seno H Konda Y Marusawa H Kanai M Nakajima T Kawashima T Nanakin A Sawabu T Uenoyama Y Sekikawa A Kawada M Suzuki K Kayahara T Fukui H Sawada M Chiba T STAT3 is constitutively activated and supports cell survival in association with survivin expression in gastric cancer cells Oncogene 2004 23 4921 4929 10.1038/sj.onc.1207606 15077160

[B13] Haura EB Turkson J Jove R Mechanisms of disease: Insights into the emerging role of signal transducers and activators of transcription in cancer Nat Clin Pract Oncol 2005 2 315 324 16264989 10.1038/ncponc0195

[B14] Kawazoe N Watabe M Masuda Y Nakajo S Nakaya K Tiam1 is involved in the regulation of bufalin-induced apoptosis in human leukemia cells Oncogene 1999 18 2413 2421 10.1038/sj.onc.1202555 10229192

[B15] Chen A Yu J Zhang L Sun Y Zhang Y Guo H Zhou Y Mitchelson K Cheng J Microarray and biochemical analysis of bufalin-induced apoptosis of HL-60 Cells BiotechnolLett 2009 31 487 494 10.1007/s10529-008-9888-x 19039527

[B16] Takai N Ueda T Nishida M Nasu K Narahara H Bufalin induces growth inhibition, cell cycle arrest and apoptosis in human endometrial and ovarian cancer cells Int J Mol Med 2008 21 637 643 18425357

[B17] Li D Qu X Hou K Zhang Y Dong Q Teng Y Zhang J Liu Y PI3K/Akt is involved in bufalin-induced apoptosis in gastric cancer cells Anticancer Drugs 2009 20 59 64 10.1097/CAD.0b013e3283160fd6 19343001

[B18] Xie CM Chan WY Yu S Zhao J Cheng CH Bufalin induces autophagy-mediated cell death in human colon cancer cells through reactive oxygen species generation and JNK activation Free Radic Biol Med 2011 51 1365 1375 10.1016/j.freeradbiomed.2011.06.016 21763418

[B19] Jing Y Watabe M Hashimoto S Nakajo S Nakaya K Cell cycle arrest and protein kinase modulating effect of bufalin on human leukemia ML1 cells Anticancer Res 1994 14 1193 2008 8074471

[B20] Watabe M Ito K Masuda Y Nakajo S Nakaya K Activation of AP-1 is required for bufalin-induced apoptosis in human leukemia U937 cells Oncogene 1998 16 779 787 10.1038/sj.onc.1201592 9488042

[B21] Sun Y Lin Y Li H Liu J Sheng X Zhang W 2,5-Hexanedione induces human ovarian granulosa cell apoptosis through BCL-2, BAX, and CASPASE-3 signaling pathways Arch Toxicol 2012 86 205 215 10.1007/s00204-011-0745-7 21901545

[B22] Yan B Research progress on Livin protein: an inhibitor of apoptosis Mol Cell Biochem 2011 357 39 45 10.1007/s11010-011-0873-7 21617971

[B23] Kitamura H Honma I Torigoe T Hariu H Asanuma H Hirohashi Y Sato E Sato N Tsukamoto T Expression of livin in renal cell carcinoma and detection of anti-livin autoantibody in patients Urology 2007 70 38 42 17656204 10.1016/j.urology.2007.03.040

[B24] Chang H Schimmer AD Livin/melanoma inhibitor of apoptosis protein as a potential therapeutic target for the treatment of malignancy Mol Cancer Ther 2007 6 24 30 10.1158/1535-7163.MCT-06-0443 17237263

[B25] Wang Q Huang Y Ni Y Wang H Hou Y siRNA targeting midkine inhibits gastric cancer cells growth and induces apoptosis involved caspase-3,8,9 activation and mitochondrial depolarization J Biomed Sci 2007 14 783 795 10.1007/s11373-007-9192-0 17665317

[B26] Pedranzini L Dechow T Berishaj M Comenzo R Zhou P Azare J Bornmann W Bromberg J Pyridone 6, a pan-Janus-activated kinase inhibitor, induces growth inhibition of multiple myeloma cells Cancer Res 2006 66 9714 9721 10.1158/0008-5472.CAN-05-4280 17018630

[B27] Yao X Zhu F Zhao Z Liu C Luo L Yin Z Arctigenin enhances chemosensitivity of cancer cells to cisplatin through inhibition of the STAT3 signaling pathway J Cell Biochem 2011 112 2837 2849 10.1002/jcb.23198 21608020

[B28] Wang Z Jin H Xu R Mei Q Fan D Triptolidedownregulates Rac1 and the JAK/STAT3 pathway and inhibits colitis-related colon cancer progression Exp Mol Med 2009 41 717 727 10.3858/emm.2009.41.10.078 19561401 PMC2772974

[B29] Kusaba T Nakayama T Yamazumi K Yakata Y Yoshizaki A Inoue K Nagayasu T Sekine I Activation of STAT3 is a marker of poor prognosis in human colorectal cancer Oncol Rep 2006 15 1445 1451 16685378

[B30] Lin L Liu A Peng Z Lin HJ Li PK Li C Lin J STAT3 Is Necessary for Proliferation and Survival in Colon Cancer-Initiating Cells Cancer Res 2011 71 7226 7237 10.1158/0008-5472.CAN-10-4660 21900397 PMC4295768

[B31] Xi RC Biao WS Gang ZZ Significant elevation of survivin and livin expression in human colorectal cancer: inverse correlation between expression and overall survival Onkologie 2011 34 428 432 10.1159/000331132 21934342

